# Mini Review: Bacterial Membrane Composition and Its Modulation in Response to Stress

**DOI:** 10.3389/fmolb.2021.634438

**Published:** 2021-05-11

**Authors:** Jessica R. Willdigg, John D. Helmann

**Affiliations:** Department of Microbiology, Cornell University, Ithaca, NY, United States

**Keywords:** bacteria, membrane, lipid, cellular envelope, antimicrobial resistance, metabolism, *Bacillus subtilis*

## Abstract

Antibiotics and other agents that perturb the synthesis or integrity of the bacterial cell envelope trigger compensatory stress responses. Focusing on *Bacillus subtilis* as a model system, this mini-review summarizes current views of membrane structure and insights into how cell envelope stress responses remodel and protect the membrane. Altering the composition and properties of the membrane and its associated proteome can protect cells against detergents, antimicrobial peptides, and pore-forming compounds while also, indirectly, contributing to resistance against compounds that affect cell wall synthesis. Many of these regulatory responses are broadly conserved, even where the details of regulation may differ, and can be important in the emergence of antibiotic resistance in clinical settings.

## Introduction: Membrane Homeostasis and Its Modulation in Response to Stress

The cell envelope is a multilayered outer barrier that protects the cell from a changing environment. Cell envelope stress responses (CESRs) are regulatory pathways that sense threats and mount a protective response, often involving modification of lipopolysaccharides (in Gram-negative bacteria), teichoic acids (Gram-positive bacteria), peptidoglycan, and the inner membrane (Helmann, 2016; Radeck et al., 2017; Mitchell and Silhavy, 2019). Here, we focus on *Bacillus subtilis* as a Gram-positive model for the role of CESRs in membrane homeostasis.

**FIGURE 1:**
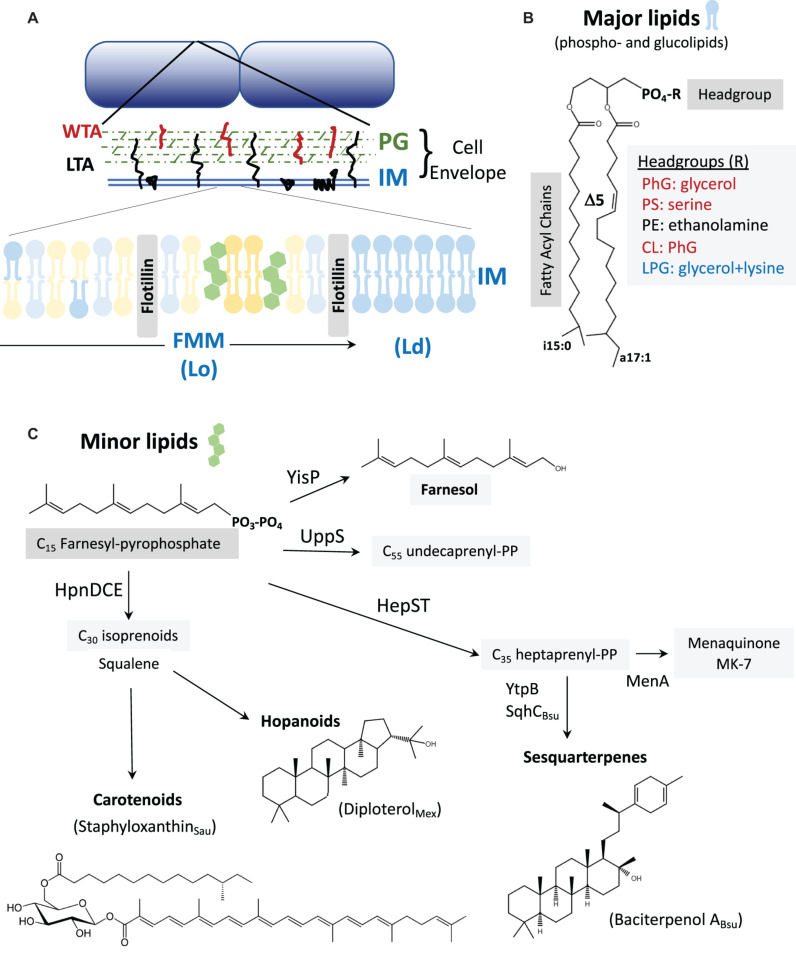
**(A)** *The cell envelope*: *Bacillus subtilis* is surrounded by a cell envelope comprised of a thick peptidoglycan (PG) layer and an inner membrane (IM). The membrane-associated lipoteichoic acid (LTA) and PG-linked wall teichoic acid (WTA) are abundant anionic polymers in the envelope (Rajagopal and Walker, 2017). The IM contains lateral microheterogeneity in the form of functional membrane microdomains (FMMs), regions of liquid-ordered (Lo) membrane together with associated proteins such as flotillins (Lopez and Koch, 2017). These are flanked by regions of higher fluidity characterized as liquid-disordered (Ld). **(B)** *Major membrane lipids*: Major membrane lipids include phospholipids and glucolipids (Nickels et al., 2017). Phospholipids (shown) vary in their FA chains, which are largely branched in *B. subtilis*. Shown here are a C_15_ iso-FA and a C_17_ Δ5 (unsaturated) anteiso-FA. Other FA chain lengths (including straight chains), and the positioning of the FA chains on the 1 and 2 positions of glycerol can vary. Variations in the phospholipid headgroups modulate surface charge (red are anionic, blue cationic, and black net neutral). Glucolipids are generally neutral lipids with one or more sugar residues in place of the phosphate shown. **(C)** *Minor membrane lipids*: Many of the minor lipids in the membrane are isoprenoids and are derived from the C_15_ intermediate farnesyl-pyrophosphate (FPP). FPP is a precursor for undecaprenyl-PP (for PG synthesis) and for the C_35_ intermediate heptaprenyl-PP. The latter is a precursor for the electron carrier menaquinone (MK-7) and sesquarterpenes including baciterpenol A and its derivatives (sporulenes) (Bosak et al., 2008; Takigawa et al., 2010; Sato et al., 2011; Sato, 2013). Two FPP can also be coupled in a multistep reaction by HpnDCE to generate C_30_ squalene (Pan et al., 2015; van der Donk, 2015), which can be processed into carotenoids [such as staphyloxanthin from *S. aureus;* (Garcia-Fernandez et al., 2017; Foster, 2019)] or cyclized by squalene-hopene cyclases to generate polycyclic compounds (hopanoids) (Saenz et al., 2015; Belin et al., 2018). In *B. subtilis*, FPP can also be dephosphorylated by YisP to generate the alcohol farnesol (Bell and Chappell, 2014; Feng et al., 2014).

The cell membrane is a dynamic, fluid mosaic comprising a lipid bilayer and associated proteins (Figure 1). In *B. subtilis*, the major lipid species are phospholipids, glucolipids, and the lipoteichoic acids (LTA) (Salzberg and Helmann, 2008; Nickels et al., 2017). The membrane proteome includes proteins for transport and signaling, as well as membrane synthesis, remodeling, and protection. As the innermost and last line of defense, the cell membrane is critical for viability. In *B. subtilis*, for example, collapsing the proton motive force activates autolysins resulting in rapid cell lysis (Jolliffe et al., 1981). Membrane-active compounds such as detergents, antimicrobial peptides, and pore-forming compounds often trigger stress responses that modify the lipidome and membrane proteome to confer resistance. Membrane stress responses can modify the cell membrane, by (i) modulating the length, branching, and saturation of the fatty acid (FA) acyl chains, (ii) altering membrane lipid composition, or (iii) synthesizing proteins that modify or protect the membrane (Table 1).

**TABLE 1:** Representative *B. subtilis* CESRs that modify the lipidome and membrane proteome^1^.

CESR	Gene(s)	Function	References
***Lipidome***			
σ^*W*^	*fabHA-fabF*	Homeoviscous adaptation; Increased anteiso FA, decreased straight chain FA	Kingston et al., 2011
DesKR	*des*	Homeoviscous adaptation; Δ-5-FA desaturase	de Mendoza, 2014
σ^*X*^, σ^*V*^	*dltABCDE*	Surface charge modification; D-alanylation of LTA, WTA; contributes to lantibiotic resistance	Cao and Helmann, 2004; Pietiainen et al., 2005; Kingston et al., 2013
σ^*X*^, σ^*V*^	*pssA-ybfM-psd*	Surface charge modification; synthesis of PE (zwitterionic lipid) from anionic phosphatidylglycerol; upregulated by 1-butanol treatment	Cao and Helmann, 2004; Vinayavekhin et al., 2015
σ^*M*^	*ytpAB*	YtpA; FA chain hydrolysis to generate lysophospholipids YtpB; initiating enzyme in sesquarterpene synthesis	Tamehiro et al., 2002; Sato et al., 2011
σ^*M*^	*ltaSa*	Alternative LTA synthase; induced in strains lacking the primary synthase (LtaS).	Eiamphungporn and Helmann, 2008; Wormann et al., 2011; Hashimoto et al., 2013
***Proteome***			
σ^*W*^	*floA floT*	FloA and FloT flotillins (SPFH family); integral membrane proteins implicated in lipid raft function; Induction of *yqeZ-floA-yqfB* operon provides resistance against sublancin.	Butcher and Helmann, 2006; Bramkamp and Lopez, 2015
σ^*W*^	*pspA*	PspA; phage shock protein A (PspA/VIPP1/IM30/ESCRT III family), membrane protection and remodeling; contributes to nisin resistance.	Kingston et al., 2013; Flores-Kim and Darwin, 2016; Manganelli and Gennaro, 2017
σ^*W*^	*yknWXYZ yfhLM*	YknWXYZ (transporter) and YfhLM provide protection against the SdpC “cannibalism toxin.” YfhL is a paralog of the SdpI immunity protein.	Butcher and Helmann, 2006; Lamsa et al., 2012; Yamada et al., 2012; Hofler et al., 2016
σ^*W*^	*ydbST*	YdbST provide protection against Amylocyclicin (cyclic lipopeptide).	Butcher and Helmann, 2006; Scholz et al., 2014
LiaRS	*liaIH*	LiaH; a PspA paralog, anchored by LiaI. Strongly induced by membrane-perturbing antimicrobials; induced by TAT protein export.	Mascher et al., 2004; Radeck et al., 2017; Bernal-Cabas et al., 2020
BceRS	*bceAB*	Prototype for flux-sensing TCS (BceRS) that integrates signals from the cognate ABC transporter (BceAB).	Fritz et al., 2015; Radeck et al., 2016; Kobras et al., 2020
LnrJK	*lrnLMN*	A flux-sensing system for induction of linearmycin and amphotericin (polyene antibiotic) resistance.	Stubbendieck and Straight, 2017; Stubbendieck et al., 2018; Revilla-Guarinos et al., 2020

*^1^ This list includes representative systems from *B. subtilis*, but does not include CESRs with related functions from other organisms.*

## The Regulation of FA Synthesis During Growth

Most bacteria utilize a type II FA synthase that catalyzes repeated cycles of acyl chain elongation (Parsons and Rock, 2013). The committed step, catalyzed by acetyl-CoA carboxylase (ACC), generates malonyl-CoA and then malonyl-ACP to serve in FA chain initiation by FabH and elongation by FabF. *B. subtilis* has two isoforms of FabH, and both preferentially synthesize branched chain FAs (BCFAs) (Choi et al., 2000; Kingston et al., 2011). Acylation of glycerol-3-phosphate by the PlsX/PlsY/PlsC acyltransferase system with long chain FAs generates phosphatidic acid, the precursor to all other phospholipids (Yao and Rock, 2013).

FapR is the key transcriptional regulator of membrane lipid synthesis in *B. subtilis* and clinically relevant pathogens such as *Staphylococcus aureus*, *Bacillus anthracis*, and *Listeria monocytogenes* (Schujman et al., 2003; Fujita et al., 2007; Albanesi et al., 2013; Machinandiarena et al., 2020), and modulates the overall rate of membrane synthesis in response to precursor availability. *B. subtilis* FapR represses genes for FA and phospholipid synthesis, and this repression is relieved by allosteric interactions with malonyl-CoA or malonyl-ACP (Schujman et al., 2006; Martinez et al., 2010).

As a branchpoint enzyme, ACC is often under complex regulation (Zhang and Rock, 2009; Salie and Thelen, 2016; Machinandiarena et al., 2020). In *B. subtilis*, ACC is regulated in part by YqhY, a conserved DUF322/Asp23 protein which is highly expressed and often encoded together with ACC subunits as part of an *accB-accC-yqhY* operon (Todter et al., 2017). The namesake, *S. aureus* Asp23, is a membrane-associated protein originally linked to alkaline shock (Petersen et al., 2020). Loss of Asp23/YqhY causes cell wall stress and poor growth (Muller et al., 2014; Todter et al., 2017). In *B. subtilis*, *yqhY* null mutants acquire suppressors that decrease ACC activity, but this selective pressure is alleviated in medium supplemented with acetate (Todter et al., 2017). We suggest that ACC-dependent depletion of acetyl-CoA may contribute to wall stress by negatively affecting synthesis of UDP-N-acetylglucosamine needed for peptidoglycan synthesis. A key challenge for future research will be to understand the precise role of YqhY/Asp23 proteins and how they control ACC activity to balance FA synthesis with other cellular needs.

## Modulating FA Composition for Homeoviscous Adaptation

Tuning of FA composition provides one way in which the cell can optimize membrane properties in response to a changing environment. Even under non-stressed conditions, *B. subtilis* membranes contain ∼7 distinct FAs varying in length from C_14_ to C_18_ (indicating the number of carbon atoms) and include both branched (∼24% iso and 66% anteiso) and straight chain (∼10%) FAs (Kingston et al., 2011). Since membrane phospholipids and glucolipids each contain 2 FA chains, the lipidome contains a complex mix of species (Figure 1B), with a preponderance containing one C_15_ and one C_17_ FA chain (Kingston et al., 2011).

Modifications of FAs are important for regulating membrane fluidity in a process known as *homeoviscous adaptation* (de Mendoza, 2014; Ernst et al., 2016). In *B. subtilis*, temperature downshift induces a FA desaturase (Des) controlled by the DesKR two-component system (TCS) (Abriata et al., 2017). Des modifies existing membrane lipids, and is thereby suited for rapid adaptation. DesK is one of the better understood TCS sensors, with both kinase and phosphatase activity (Abriata et al., 2017; Fernandez et al., 2019). DesK lacks an extracellular sensor domain, but has multiple transmembrane segments that sense changes in the membrane physical state. DesK phosphorylates the DesR response regulator, which induces *des*, encoding a FA Δ5 desaturase (Altabe et al., 2003). The resultant unsaturated FAs increase bilayer fluidity, which restores DesK phosphatase activity in a negative feedback loop (de Mendoza, 2014). Longer term adaptation to low temperatures relies on an isoleucine-dependent switch to primarily anteiso-FAs (Weber et al., 2001). Since anteiso-FAs perturb the lateral interactions between adjacent lipids to a greater extent than iso-FAs (Figure 1B), this shift increases membrane fluidity (Kingston et al., 2011). This shift may result from a cold-dependent change in FabH activity (Beranova et al., 2008; Saunders et al., 2016).

Membranes must also adapt to conditions that increase fluidity. In *B. subtilis*, the ECF σ factor σ^*W*^ is activated by detergents, antibiotics, and bacteriocins active on the membrane (Cao et al., 2002b; Pietiainen et al., 2005; Butcher and Helmann, 2006; Helmann, 2006, 2016). A σ^*W*^ promoter within the *fabHA-fabF* operon plays a major role in homeoviscous adaptation (Kingston et al., 2011). Activation of σ^*W*^ leads to a decrease in FabHA levels, resulting in increased reliance on FabHB and an increase in straight chain FAs (from ∼10 to 30%). Elevated expression of the FabF elongation enzyme leads to increased FA chain length. The combined effect is a membrane with longer acyl chains and less BCFA. This increased membrane rigidity serves to protect cells against detergents and antimicrobial peptides (Kingston et al., 2011). The activation of σ^*W*^ is controlled by regulated proteolysis of its membrane-bound anti-σ^*W*^ factor (RsiW) (Schobel et al., 2004; Ellermeier and Losick, 2006; Devkota et al., 2017). However, the mechanisms by which membrane stressors trigger σ^*W*^ activation remain unclear.

To better understand the role of FA heterogeneity in controlling membrane properties, it would be desirable to study bacteria with chemically simple membranes. This has been achieved in *B. subtilis* by feeding exogenous FAs to cells with *de novo* FA synthesis blocked by cerulenin and a mutation to inhibit FA degradation (Nickels et al., 2020). Growth can be rescued with only two FA species: a straight-chain C_16_ FA (high melting) and an anteiso C_15_ FA (low melting). Even with only these two FA species, four distinct arrangements are possible upon acylation of glycerol-3-phosphate to generate phosphatidic acid. Cells compensate for this reduced FA complexity by altering the distribution of phospholipid headgroups, a modest induction of the DesRK system, apparent downregulation of the σ^*W*^ stress response, and an increase in isoprenoid lipids (Nickels et al., 2020). These results highlight the remarkable adaptability of bacterial membranes, and the interconnection between diverse stress responses.

## Overview of Membrane Lipid Composition and Synthesis

One of the persistent challenges in membrane biology is to define the roles of the diverse constituent lipids (Sohlenkamp and Geiger, 2016; Dowhan et al., 2019; Chwastek et al., 2020). Although membranes have a complex and adaptable composition (the lipidome), cells are remarkably resilient to genetic alterations that remove lipid species. Because of its single membrane and ease of genetic manipulation, *B. subtilis* presents an attractive model system (Nickels et al., 2017). The *B. subtili*s lipidome comprises ∼70% phospholipids and ∼30% neutral glucolipids. The major phospholipids are phosphatidylglycerol (PhG) and phosphatidylethanolamine (PE), with minor contributions from cardiolipin and lysylphosphatidylglycerol (LPG). Variations in phospholipid headgroup size and charge modulate membrane properties (Figure 1B). Membranes also contain LTA anchored to neutral glucolipids, which together with peptidoglycan-linked wall teichoic acid (WTA) can account for up to 60% of the dry weight of the cell wall (Rajagopal and Walker, 2017; Sumrall et al., 2020). However, LTA fractionates with the wall during membrane lipid extraction, and is often not considered in lipidome measurements.

The only essential phospholipid in *B. subtilis* is PhG. Remarkably, the membrane can be simplified to contain close to 100% PhG with no glucolipids. Despite a greatly simplified membrane, such mutants can grow rapidly, albeit with a highly abnormal coiled filament morphology (Salzberg and Helmann, 2008). Genetic perturbations of membrane composition can lead to resistance to cationic antimicrobial peptides (CAMP). For example, gain-of-function mutations in *mprF*, encoding the LPG synthase/flippase, can confer daptomycin resistance possibly by reducing surface charge (Ernst et al., 2018; Ernst and Peschel, 2019). Consistently, *mprF* null mutants have increased daptomycin sensitivity and overexpression decreases sensitivity in *B. subtilis* (Hachmann et al., 2009). Daptomycin resistance also results from *pgsA* mutations that decrease PhG levels (Hachmann et al., 2011; Peleg et al., 2012).

In addition to the dominant phospholipids and glucolipids, membranes contain numerous other lipid species. Most prominent are the isoprenoid lipids synthesized by polymerization of C_5_ isoprene units (Figure 1C). The key intermediate farnesyl-PP (C_15_) can be joined (head-to-head) to generate squalene (C_30_) (Pan et al., 2015; van der Donk, 2015), a precursor of cholesterol and other sterols in eukaryotes and of structurally related hopanoid lipids in many bacteria. One major hopanoid is diploterol (Figure 1C), with five fused rings that can be further modified in a variety of ways (Belin et al., 2018). Farnesyl-PP can also be extended by UppS, which sequentially adds eight isopentenyl units to generate undecaprenyl-PP, the C_55_ carrier lipid that supports cell wall synthesis (Figure 1C). Alternatively, the HepST complex can extend farnesyl-PP to generate heptaprenyl (C_35_)-PP, an isoprenoid used as a lipid anchor for menaquinone (MK-7), the electron carrier for respiration. In *B. subtilis*, this same precursor can be processed to polycyclic C_35_-sesquarterpenoids, which may be functionally similar to C_30_ hopanoids (Bosak et al., 2008; Takigawa et al., 2010; Sato et al., 2011; Sato, 2013). This process is initiated by YtpB, which generates tetraprenyl-β-curcumene, and then SqhC (a homolog of squalene-hopene cyclases) to generate the C_35_ tetracyclic product known as baciterpenol A (Sato, 2013). Although initially described in spores, and named “sporulenes” (Bosak et al., 2008), these sesquarterpenoids are found in vegetative cells (Takigawa et al., 2010). Finally, heptaprenyl-PP can be coupled to glycerol-1-phosphate by PcrB, and then further processed by an unidentified phosphatase and the YvoF acetyltranferase to generate an ether linked lipid of unknown function (Linde et al., 2016).

### Lateral Heterogeneity and Functional Membrane Microdomains

In eukaryotes, cholesterol is associated with the generation of functional membrane microdomains (FMM), also called lipid rafts. These regions have relatively low membrane fluidity (a liquid-ordered, or Lo phase) and are associated with flotillins. *B. subtilis* also encodes flotillin homologs, regulated by σ^*W*^ (Huang et al., 1999; Wiegert et al., 2001). These proteins, subsequently renamed FloA and FloT, are implicated in FMM formation (Figure 1A). The notion of FMMs in bacteria received strong impetus from the finding that *yisP* mutants, lacking a putative squalene synthase, was defective in biofilm formation (Lopez and Kolter, 2010). Together with the finding of a punctate localization for FloT, and chemical inhibition studies with compounds that affect sterol synthesis, this led to the proposal that bacteria harbor FMMs (Bramkamp and Lopez, 2015; Wagner et al., 2017). However, subsequent work revealed that YisP is a farnesyl-PP phosphatase that generates farnesol (Figure 1C), rather than squalene (Feng et al., 2014). Moreover, farnesol itself complements the biofilm defect of the *yisP* mutant, suggesting that this long chain alcohol may have an ordering effect on FMMs provided in other systems by hopanoids or carotenoids (Bell and Chappell, 2014; Feng et al., 2014). *B. subtilis* FMMs are enriched in flotillins (FloA and FloT) and their associated signaling complexes, with FMM formation apparently stabilized by farnesol (YisP product). No role for the C_35_ isoprenoid lipids has yet been demonstrated in biofilm formation (Lopez and Kolter, 2010) or in FMM formation or function.

Lateral heterogeneity, including FMMs, is likely a feature of most bacterial membranes. However, the lipid species that are required to form FMMs are still poorly understood, but likely include carotenoids, hopanoids, and other polycyclic isoprenoid lipids (Lopez and Koch, 2017). Hopanoids are structurally diverse and fulfill a broad range of functions in bacterial membranes (Belin et al., 2018). The hopanoid diplopterol (Figure 1C) orders saturated lipids and glycolipids in the outer membrane of *Methylobacterium extorquens*, and deficient mutants are impaired in multidrug transport (Saenz et al., 2015). Hopanoids and other polycyclic isoprenoids are present in many Gram-positive bacteria as well, suggestive of a role in the plasma membrane. In methicillin-resistant *S. aureus*, the carotenoid staphyloxanthin (Figure 1C) colocalizes in FMMs with FloA, and disruption of these domains with isoprenoid synthesis inhibitors interferes with the function of the penicillin-binding protein required for β-lactam resistance (PBP2a) (Garcia-Fernandez et al., 2017; Foster, 2019). The formation and function of FMMs, in both the inner (plasma) and outer membrane, remains an important area for future research.

## Cell Envelope Stress Responses That Modulate Lipid Composition

Bacteria generally have a negatively charged membrane, which contributes to their susceptibility to CAMPs, bacteriocins, and antimicrobials. In *B. subtilis*, membrane composition and properties are regulated by ECF σ factors (Eiamphungporn and Helmann, 2008; Kingston et al., 2013; Helmann, 2016). Because of their overlapping activation and promoter recognition properties, these CESRs are intertwined and referred to as an σ^*ECF*^ stress response (Mascher et al., 2007). In *B. subtilis*, activation of σ^*X*^ reduces the net negative charge of the membrane by increasing zwitterionic PE levels (Cao and Helmann, 2004; Ho and Ellermeier, 2019). The net negative charge of the cell wall can be further reduced by D-alanylation of teichoic acids, activated by σ^*X*^ (Cao and Helmann, 2004; Ho and Ellermeier, 2019) and σ^*V*^, a lysozyme-responsive CESR (Guariglia-Oropeza and Helmann, 2011; Ho et al., 2011; Ho and Ellermeier, 2019). In *S. aureus*, surface membrane charge is modified by the induction of *mprF* by the GraRS TCS, thereby increasing LPG levels (Falord et al., 2011; Yang et al., 2012). In *B. anthracis*, the membrane-active compound targocil activates the EdsRS TCS, which induces expression of a cardiolipin synthase (Laut et al., 2020). Thus, many different stimuli can trigger changes in the membrane lipidome.

*Bacillus subtilis* σ^*ECF*^ factors also control other membrane-related functions, although the effects are not yet understood. For example, σ^*M*^ activates the *ytpAB* operon. The YtpA lysophospholipase cleaves FAs from the 2 position of phospholipids resulting in a lysophospholipid (bacilysocin) suggested to function as an antibiotic (Tamehiro et al., 2002). However, it is unclear if bacilysocin is ever released at levels sufficient to serve as an antibiotic, and it may instead modify membrane properties or be an intermediate in lipid remodeling. As noted above, YtpB initiates synthesis of baciterpenol (Figure 1C; Bosak et al., 2008; Sato et al., 2011; Sato, 2013). Genetic studies have revealed only modest phenotypes for *ytpAB* mutants, including effects on antibiotic sensitivity, sporulation, and germination (Kingston et al., 2014; Sayer et al., 2019). In the case of *ytpB*, the observed phenotype (bacitracin sensitivity) was due to the accumulation of the substrate (heptaprenyl-PP) rather than a loss of baciterpenol (Kingston et al., 2014).

Genetic perturbations of membrane composition can also trigger CESRs. For example, deletion of LTA synthases induces σ^*ECF*^ factors. An *ltaS* mutation upregulates σ^*M*^, which then activates expression of the alternate LTA synthase LtaSa. The absence of both *ltaS* and *ltaSa* leads to activation of additional σ^*ECF*^ factors (Hashimoto et al., 2013). The depletion of PhG, a building block of LTA, also activates σ^*M*^ and to a lesser extent σ^*V*^ (Hashimoto et al., 2009; Seki et al., 2019). The effects of mutations that affect glucolipids have been particularly challenging to understand. Glucolipids produced by UgtP are important membrane lipids and also serve as the lipid anchor of LTA. *ugtP* mutants lacking glucolipids are shorter and rounder, have abnormal localization of MreB, and altered assembly of FtsZ (Weart et al., 2007). Whether this abnormal morphology is due, in part, to the loss of glucolipids is unclear (Matsuoka, 2018). Mutation of *ugtP* activates a σ^*ECF*^ stress response and can be suppressed by production of monoglycosyldiacylglycerol (MGlcDG) using a heterologous synthase. Since this product does not function as an LTA anchor lipid, this suggests that it is the loss of glucolipids that induces the σ^*ECF*^ response (Matsuoka et al., 2016). The mechanistic basis for activation of σ^*ECF*^ factors in the absence of glucolipids is unclear, but at least for σ^*V*^ does not require intramembrane proteolysis of the anti-σ factor (Seki et al., 2019). One hypothesis is that glucolipids might regulate folding and function of intramembrane proteins (Matsuoka, 2018).

## Cell Envelope Stress Responses That Function Through Membrane Proteins

In addition to modulating lipid composition, CESRs also induce proteins that function in membrane protection and remodeling. In *B. subtilis*, these proteins include two flotillin homologs (FloA, FloT), two members of the phage shock protein family (LiaH, PspA), as well as antibiotic specific detoxification modules. The roles of these proteins in stabilizing and repairing the membrane are increasingly appreciated, although the precise mechanisms remain controversial.

### Flotillins and Modulation of Membrane Fluidity

Flotillins are members of the widely conserved stomatin, prohibitin, flotillin, and HflK/C (SPFH) domain proteins. Flotillins localize to FMMs and are thought to be required for FMM function. In *S. aureus*, FloA colocalizes with staphyloxanthin in FMMs (Garcia-Fernandez et al., 2017; Foster, 2019). In other systems, flotillins and FMMs are associated with flagellar function and chemotaxis (Padilla-Vaca et al., 2019; Takekawa et al., 2019), type VII secretion (Mielich-Suss et al., 2017), signaling (Wagner et al., 2017), and interaction with the host during infection (Hutton et al., 2017). Ongoing efforts strive to track the mobility, oligomerization state, and interaction partners of flotillins in living cells.

*Bacillus subtilis* FloA and FloT are oligomeric, integral membrane proteins implicated in the formation of FMMs (Lopez and Kolter, 2010; Bach and Bramkamp, 2013; Bramkamp and Lopez, 2015; Lopez and Koch, 2017), and regulated by σ^*W*^ (Huang et al., 1999; Cao et al., 2002a). FloA and FloT are thought to help partition the membrane into low fluidity FMM regions that are spatially distinct from more fluid regions. A direct role for flotillins in FMM formation has been challenged, however, since *B. subtilis* FloA and FloT do not always colocalize, and form separated foci of ∼100 nm in diameter that appear spatially distinct from FMMs (Dempwolff et al., 2016). Counter-intuitively, flotillins appear to be required for regions of increased fluidity (RIFs), which are the counterpart to the FMMs. A lack of flotillins leads to a decrease in membrane fluidity and a concomitant reduction in activity of the MreB-directed elongasome complex that synthesizes peptidoglycan. This loss of membrane fluidity can be chemically complemented with fluidizing agents such as benzoyl alcohol (Zielinska et al., 2020).

Flotillins also functionally interact with DynA, a constitutively expressed dynamin homolog (Dempwolff et al., 2012; Dempwolff and Graumann, 2014). Dynamins are membrane-associated GTPases implicated in membrane remodeling, fusion and fission, and lipid mixing (Guo and Bramkamp, 2019). DynA may help repair damaged membrane regions, and contribute to resistance against antibiotics that bind membrane components, including nisin, bacitracin, and daptomycin (Sawant et al., 2016). Our understanding of flotillins and dynamins, and their roles in bacterial physiology is still incomplete and rapidly evolving.

### Phage-Shock Proteins Protect Membrane Integrity

Cell envelope stress responses also support membrane stability through induction of PspA proteins, including two paralogs in *B. subtilis*: PspA an LiaH. Originally defined as part of the phage-shock protein response in *Escherichia coli* (Kobayashi et al., 2007; Flores-Kim and Darwin, 2016), PspA proteins comprise a conserved family including the vesicle-inducing protein in plastids (VIPP1/IM30) and mammalian ESCRT III (Thurotte et al., 2017; Liu et al., 2020). PspA proteins have a conserved N-terminal amphipathic helix required for membrane binding (McDonald et al., 2015, 2017), which seems to depend on anionic lipid content and regions with unfavorable packing geometries creating stored curvature elastic stress (McDonald et al., 2015). Structural studies reveal that VIPP1 forms oligomeric rings of various symmetries that stack together to form domes (Saur et al., 2017; Gupta et al., 2020). These rings are dynamic, and are thought to stabilize membranes during budding, tubulation, and fusion (Thurotte et al., 2017; Gutu et al., 2018; Junglas and Schneider, 2018). However, the role of these oligomeric structures has been questioned (Siebenaller et al., 2019). An alternative model suggests that these rings dissociate, and the resultant intrinsically disordered monomers interact with the membrane surface to form a protective protein “carpet” to stabilize the membrane and suppress proton leakage (Junglas et al., 2020).

Although PspA proteins are assumed to function in membrane protection and repair, their regulation differs markedly (Manganelli and Gennaro, 2017). *B. subtilis* PspA is regulated by σ^*W*^ (Wiegert et al., 2001; Cao et al., 2002a), whereas the paralog LiaH is regulated by the LiaRS TCS (Mascher et al., 2004; Jordan et al., 2006). Both paralogs localize to the membrane in response to stress and protect against membrane-damaging antibiotics (Wolf et al., 2010; Kingston et al., 2013; Dominguez-Escobar et al., 2014; Popp et al., 2020). In the case of LiaH, membrane association is mediated by interaction with the integral membrane protein LiaI (Dominguez-Escobar et al., 2014). LiaH may also protect the membrane against proton leakage during the export of proteins through the twin-arginine translocation (TAT) system (Hou et al., 2018; Bernal-Cabas et al., 2020). While the *B. subtilis* LiaRS regulon is rather limited in scope (Jordan et al., 2006; Wolf et al., 2010), LiaRS orthologs (e.g., *S. aureus* VraRS) play an important role in stress resistance in many Gram-positive pathogens, and mutations in these regulators are associated with clinical antibiotic resistance (Tran et al., 2016). In *Mycobacterium tuberculosis*, the PspA ortholog is also under control of the ECF σ factor σ^*E*^ (Datta et al., 2015), whereas *E. coli pspA* requires the σ^54^ RNAP and PspF activator (Joly et al., 2010; Flores-Kim and Darwin, 2016). A common theme in these systems is that PspA-like proteins are often regulated by a specific CESR; they can accumulate to high levels in stressed cells, and they seem to protect the membrane against disruptions that can dissipate the proton gradient (Manganelli and Gennaro, 2017).

### Antibiotic Specific Detoxification Modules

*Bacillus subtilis*, like many soil bacteria, can synthesize a wide range of antimicrobial compounds and also encodes diverse resistance mechanisms (Stein, 2005; Caulier et al., 2019). Many antimicrobial peptides induce the *B. subtilis* LiaRS stress response that protects cells through induction of LiaH. Induction of σ^*W*^ also leads to expression of the SppA membrane-localized protease and its regulatory protein SppI, which function to clear the membrane of embedded peptides to protect against lantibiotics (Kingston et al., 2013; Henriques et al., 2020). Other antimicrobial peptides induce specific detoxification machinery, often including ABC transporters that either export the peptide antibiotic or disassemble membrane-bound peptide complexes (Staron et al., 2011; Dintner et al., 2014).

A prototype for such systems is the BceRS TCS, which regulates the bacitracin-specific induction of the BceAB ABC transporter (Radeck et al., 2016; Piepenbreier et al., 2020). Bacitracin is a peptide antibiotic made by *Bacillus* spp. that inhibits cell wall synthesis by binding to undecaprenyl-PP. The BceAB system appears to act in disassembly of bacitracin complexes to confer resistance (Kobras et al., 2020). In addition, BceAB interacts with the BceRS TCS to allow sensing of bacitracin (Ohki et al., 2003; Dintner et al., 2014; Fritz et al., 2015; Koh et al., 2020). The BceRS-AB system provides a first line of defense against bacitracin, with higher levels of antibiotic activating the protective responses mediated by the LiaRS and σ^*ECF*^ regulons (Radeck et al., 2016). The detailed study of the *B. subtilis* bacitracin stress response has provided lessons relevant to the understanding of other antibiotic detoxification modules. Similar genetic modules, encoding both TCS and ABC transporter/sensors have been described for several other antimicrobial peptides (Revilla-Guarinos et al., 2014). Since induction can be quite specific, these systems provide a basis for antibiotic-inducible gene expression systems (Wolf and Mascher, 2016).

*Bacillus subtilis* also encodes and responds to many other secondary metabolites that can induce membrane stress (Caulier et al., 2019). For example, the toxic peptide YydF^∗^ is encoded by the *yydFGHIJ* operon, together with a radical-SAM epimerase (YydG), protease (YydH), and ABC transporter (YydIJ). Transposon insertions in the presumptive efflux pump lead to the upregulation of the LiaRS stress system (Butcher et al., 2007). Subsequent studies revealed that YydF is post-translationally processed to convert two L-amino acids to D-amino acids (Benjdia et al., 2017). The resulting epipeptide, YydF^∗^, induces LiaRS-regulated LiaH and the FloT flotillin (Popp et al., 2020). The modified YydF^∗^ peptide kills *B. subtilis* cells by dissipating the membrane potential via membrane permeabilization. The associated concomitant decrease in membrane fluidity together with increased membrane permeabilization induces *liaIH* (Popp et al., 2020). YydF^∗^ peptides are likely synthesized by a variety of Gram-positive organisms including *Enterococcus*, *Staphylococcus*, and *Streptococcus* spp. as well as members of the human microbiome (Benjdia et al., 2017).

*Bacillus subtilis* also has CESRs induced by polyketide and polyene-type antimicrobials. For example, *Streptomyces* spp. produce linear polyketides (linearmycins) that depolarize the membrane (Stubbendieck and Straight, 2015, 2017; Stubbendieck et al., 2018). Linearmycins strongly activate the LnrJK TCS that regulates an ABC transporter, LnrLMN (Stubbendieck and Straight, 2017; Revilla-Guarinos et al., 2020). This ABC transporter also provides resistance against other polyenes, including the anti-fungal amphotericin (Revilla-Guarinos et al., 2020).

## Outlook

Here we provide a brief overview of the diverse ways in which CESRs help modify and protect the membrane in response to environmental threats (Table 1). This is a rapidly evolving field, and the impact of membrane composition on cell physiology is still mysterious. We have much to learn about the synthesis and roles of minor lipids (sesquarterpenes, ether lipids, lysophospholipids). There is a growing need to reconcile current models of lipid rafts, and the role that isoprenoid lipids and flotillins play in their formation. The activities of the VIPP1/IM30/PspA family of proteins in membrane repair and protection, and in particular the specific role of different oligomeric states, are still debated. Finally, the mechanisms by which diverse CESRs sense membrane perturbations are largely unknown, although considerable progress has been made in the specific cases of the DesK sensor kinase (Abriata et al., 2017), flux-sensing by peptide detoxification modules (Koh et al., 2020), and the lysozyme-mediated induction of the σ^*V*^ protein (Ho and Ellermeier, 2019). The overall picture is of the cell membrane as a complex and adaptable assemblage of many different lipid and protein species that still has many secrets to reveal.

## Author Contributions

Both authors listed have made a substantial, direct and intellectual contribution to the work, and approved it for publication.

## Conflict of Interest

The authors declare that the research was conducted in the absence of any commercial or financial relationships that could be construed as a potential conflict of interest.
